# High prevalence of CTX-M-1 and CTX-M-15 extended-spectrum β-lactamase-producing *Escherichia coli* in retail imported frozen beef in Saudi Arabia

**DOI:** 10.14202/vetworld.2026.569-579

**Published:** 2026-02-17

**Authors:** Lamya Zohair Yamani, Nasreldin Elhadi

**Affiliations:** Department of Clinical Laboratory Sciences, College of Applied Medical Sciences, Imam Abdulrahman Bin Faisal University, Box 2435, 31441 Dammam, Kingdom of Saudi Arabia

**Keywords:** antimicrobial resistance, beef safety, *Escherichia coli*, extended-spectrum beta-lactamase, foodborne pathogens, imported meat, multidrug resistance, One Health

## Abstract

**Background and Aim:**

The global expansion of antimicrobial resistance (AMR), particularly due to extended-spectrum β-lactamase (ESBL)-producing *Escherichia coli*, represents a critical threat to food safety and public health. Imported meat products are increasingly recognized as potential vehicles for the transboundary dissemination of multidrug-resistant (MDR) bacteria. However, data on the occurrence and molecular characteristics of ESBL-producing *E. coli* in imported frozen beef in Saudi Arabia remain limited. This study aimed to determine the prevalence, AMR profiles, and ESBL gene distribution among *E. coli* isolated from retail imported frozen beef.

**Materials and Methods:**

A total of 78 imported frozen boneless beef samples were collected from retail shops and supermarkets in Dammam and Al Khobar, Eastern Province, Saudi Arabia. *E. coli* isolation was performed using Enterobacteriaceae enrichment broth followed by CHROMagar™ *E. coli*. Presumptive isolates were screened on CHROMagar™ ESBL and subjected to antimicrobial susceptibility testing against 21 antimicrobials using the disc diffusion method. Phenotypic confirmation of ESBL production was conducted using E-test ESBL strips. Molecular detection of ESBL-encoding genes (*bla*_TEM_, bla_SHV_, *bla*_CTX-M-1_, *bla*_CTX-M-9_, and *bla*_CTX-M-15_) was performed by polymerase chain reaction.

**Results:**

From 78 beef samples, 390 *E. coli* isolates were recovered, of which 361 (92.5%) were presumptive ESBL producers on CHROMagar™ ESBL. Phenotypic confirmation showed that 319/361 (88.3%) isolates were ESBL-producing *E. coli*. Molecular analysis detected β-lactamase genes in 324/361 (89.7%) isolates, with *bla*_CTX-M-1_ and *bla*_CTX-M-15_ each identified in 87.5% of isolates. High resistance rates were observed to ampicillin (99.4%), cephalothin (97.2%), cephalexin (91.4%), ceftriaxone (81.9%), and cefotaxime (73.9%). MDR was detected in 97.2% of isolates.

**Conclusion:**

Retail imported frozen beef in Saudi Arabia harbors an exceptionally high burden of MDR ESBL-producing *E. coli*, predominantly driven by CTX-M-1 and CTX-M-15 enzymes. These findings indicate that international beef supply chains may act as significant reservoirs for high-risk ESBL determinants, underscoring the need for strengthened surveillance, regulatory control, and One Health-based interventions to limit foodborne dissemination of AMR.

## INTRODUCTION

Global health challenges have increasingly emerged as a consequence of rising antimicrobial resistance (AMR). Extended-spectrum β-lactamase-producing *Escherichia coli* (ESBL-producing *E. coli*) has been closely linked to this global threat [1]. *E. coli* has been isolated not only from clinical environments but also from food products imported from endemic regions. This bacterium is capable of hydrolyzing a broad range of β-lactam antibiotics, including penicillins and third-generation cephalosporins [2,3]. This hydrolytic activity disrupts the amide bonds within the four-membered β-lactam ring, thereby inactivating these antimicrobial agents [4].

β-lactamases are classified into four major groups based on molecular size and structural similarities within their active amino acid sites. Three groups belong to the serine β-lactamase classes A, C, and D, whereas the fourth group comprises metallo-β-lactamases (class B). ESBLs fall predominantly within class A and represent the most prevalent group [5]. These enzymes confer resistance to third-generation cephalosporins, such as cefotaxime, ceftriaxone, cefixime, and ceftazidime, which are critically important in both human and veterinary medicine. During the 1980s, a marked increase in ESBL prevalence was documented, largely associated with SHV and TEM variants that dominated early reports. However, following the first identification in Germany in 1989 of cefotaxime-resistant *E. coli* strains unrelated to TEM or SHV types, the cefotaxime-Munich (CTX-M) group emerged as the most widespread ESBL family worldwide and is now considered a major cause of infections in humans and animals within the Enterobacteriaceae [6, 7].

CTX-M enzymes originated from the mobilization of chromosomal β-lactamase genes in Kluyvera species, which subsequently became incorporated into mobile plasmids. Further diversification of CTX-M variants has occurred through point mutations driven by selective advantages under antibiotic pressure [8]. These enzymes act as natural cephalosporinases, exhibiting high activity against cefotaxime and ceftriaxone, and conferring resistance to other extended-spectrum cephalosporins, including cefepime and ceftazidime [9]. Although CTX-M genes are most commonly located on transferable conjugative plasmids flanked by insertion sequences that enhance mobility and expression, chromosomal integration has been reported with increasing frequency [10]. Based on amino acid sequence homology, CTX-M enzymes are classified into five phylogenetic families, each named after the first identified member [9], with more than 230 variants described to date [11].

The CTX-M family, particularly the CTX-M-1 and CTX-M-15 types, has become the dominant ESBL group globally, largely replacing earlier TEM and SHV types [12, 13]. These enzymes have been identified in both hospital-acquired and community-associated *E. coli* isolates [14]. ESBL-producing *E. coli* has also been recovered from various food products, including chicken, fish, and beef, often as a result of antibiotic use and misuse in animal production for disease control and growth promotion [15,16]. Imported meat products are increasingly recognized as important reservoirs and transmission routes for multidrug-resistant (MDR) bacteria, facilitating the silent spread of resistance genes into the human microbiota [17, 18]. The dissemination of these determinants is frequently mediated by mobile genetic elements, such as plasmids carrying resistance genes to multiple antimicrobial classes, posing a significant threat to public health [19].

Despite the growing global concern regarding AMR, data on ESBL-producing *E. coli* associated with imported meat products remain fragmented, particularly in the Middle East. Existing studies have largely focused on clinical isolates or locally produced foods, while systematic investigations addressing the prevalence, resistance profiles, and molecular characteristics of ESBL-producing *E. coli* in imported frozen beef are scarce. Moreover, limited information is available on the dominance and co-occurrence of key ESBL determinants, particularly CTX-M-1 and CTX-M-15, in retail beef products entering Saudi Arabia. The lack of integrated phenotypic and genotypic surveillance data hampers accurate risk assessment of MDR *E. coli* dissemination through international food supply chains and constrains the development of evidence-based food safety and AMR mitigation strategies within a One Health framework.

The present study aimed to investigate the occurrence of ESBL-producing *E. coli* in retail imported frozen beef marketed in the Eastern Province of Saudi Arabia. Specifically, this study sought to determine the prevalence of ESBL and MDR phenotypes among *E. coli* isolates, characterize their antimicrobial susceptibility patterns, and identify the distribution of major ESBL-encoding genes using molecular methods. By integrating phenotypic screening with genotypic confirmation, this work aims to provide baseline surveillance data on foodborne ESBL-producing *E. coli* and to clarify the potential role of imported frozen beef as a reservoir for high-risk AMR determinants.

## MATERIALS AND METHODS

### Ethical approval

This study was based exclusively on the examination of commercially available imported frozen beef samples, and no experiments involving live animals were conducted. Ethical review and approval were obtained from the Institutional Review Board, Imam Abdulrahman Bin Faisal University (IRB No. IRB 2014-04-189).

### Study period and location

This study was conducted from April 2014 to April 2016 at Microbiology Research Laboratory, Department of Clinical Laboratory Sciences, Faculty of Applied Medical Sciences, Imam Abdulrahman Bin Faisal University. All *E. coli* isolates were stored in –80°C fridge in trypticase soy broth (TSB) containing 20% glycerol at Microbiology Research Laboratory. Between 2020 and 2021, all *E. coli* isolates stored in -800C fridge were analyzed using PCR to detect ESBL determinants genes.

### Sample collection

A total of 78 imported frozen boneless beef samples were collected from retail shops and supermarkets located in Dammam and Al Khobar in the Eastern Province of Saudi Arabia. Samples were collected to investigate the presence of ESBL-producing *E. coli* and MDR among *E. coli* isolates. All samples were aseptically placed in sterile containers, maintained under ice, and transported to the microbiology research laboratories for microbiological analysis and genotypic characterization.

### Isolation and identification of E. coli

*E. coli* was isolated from frozen beef samples using Enterobacteriaceae enrichment (EE) broth (CM013, Oxoid, Basingstoke, UK) and CHROMagar™ *E. coli* (CHROMagar, Paris, France). Briefly, 25 g of each beef sample was aseptically weighed and transferred into a sterile stomacher bag containing 225 mL of EE broth [20]. Samples were homogenized for 2 min using a laboratory blender (Seward Stomacher 400 Circulator, Seward, Worthing, UK) and incubated at 37°C for 24 h.

Following enrichment, 0.1 mL of the broth culture was streaked onto CHROMagar™ *E. coli* and incubated at 37°C for 24 h. Typical blue colonies suggestive of *E. coli* were selected and confirmed using API 20E identification strips (bioMérieux, Marcy-l’Étoile, France) along with standard oxidase and indole biochemical tests. Confirmed isolates were subsequently subcultured onto CHROMagar™ ESBL (CHROMagar, Paris, France). Colonies exhibiting dark pink to red coloration were presumptively identified as ESBL-producing *E. coli* and subjected to further antimicrobial susceptibility testing (AST). Phenotypic confirmation of ESBL production was performed using E-test ESBL strips, while genotypic confirmation was carried out by polymerase chain reaction (PCR).

### AST

All *E. coli* isolates recovered on CHROMagar™ ESBL were tested for antimicrobial susceptibility using the disk diffusion method according to Clinical and Laboratory Standards Institute guidelines [21]. The antimicrobials tested included ciprofloxacin (CIP), cefotaxime (CTX), ceftazidime (CAZ), ceftriaxone (CRO), cephalexin (CFX), cefoxitin (FOX), cephalothin (KF), ampicillin (AM), amoxicillin/clavulanic acid (AUG), piperacillin (PIP), nalidixic acid (NA), norfloxacin (NOR), amikacin (AK), gentamicin (GM), kanamycin (K), tobramycin (TN), nitrofurantoin (FM), aztreonam (ATM), chloramphenicol (C), trimethoprim/sulfametho-xazole (SXT), and tetracycline (TE).

*E. coli* American Type Culture Collection 25922 was used as the reference strain for quality control. Results were interpreted according to CLSI criteria [21]. MDR was defined as non-susceptibility to at least one agent in three or more antimicrobial classes, following the criteria described by Magiorakos et al. [22].

### Phenotypic screening for ESBL

Phenotypic confirmation of ESBL production was conducted using E-test ESBL strips (bioMérieux, Marcy-l’Étoile, France) after completion of AST. The E-test ESBL strips were used to determine minimum inhibitory concentrations (MICs) for ceftazidime/ceftazidime + clavulanic acid (TZ/TZL) and cefotaxime/ cefotaxime + clavulanic acid (CT/CTL). The strips generated concentration gradients for TZ (0.5–32 mg/L), TZ/TZL (0.064–4 mg/L plus 4 mg/L clavulanic acid), CT (0.25–16 mg/L), and CT/CTL (0.064–4 mg/L plus 4 mg/L clavulanic acid).

All selected *E. coli* isolates were tested according to the manufacturer’s instructions. ESBL production was confirmed when MIC values met the criteria: CT ≥ 0.5 mg/L with CT/CTL ≥ 8, and TZ ≥ 1 mg/L with TZ/TZL ≥ 8. ESBL activity was also inferred from the presence of “phantom” zones or deformation of inhibition ellipses. Results exceeding the measurable range of the strips were classified as non-determinable (ND).

### DNA extraction and genotyping of β-lactamase genes

Genomic DNA was extracted using a boiling method as previously described by Yamani and Elhadi [23]. Briefly, 1.0 mL of an overnight *E. coli* culture grown in Luria–Bertani broth was centrifuged at 10,000 × *g* for 2 min. The pellet was resuspended in 1.0 mL of sterile distilled water and boiled at 100°C for 15 min, followed by centrifugation at 12,000 × *g* for 5 min. The supernatant containing DNA was transferred to a fresh tube and stored at −20°C until PCR analysis.

PCR was performed to detect *bla*_CTX-M_, *bla*_CTX-M-1_, *bla*_CTX-M-15_, *bla*_CTX-M-9_, *bla*_TEM_, and *bla*_SHV_ genes using specific oligonucleotide primers as previously described [24–29]. Amplification reactions were carried out using GoTaq® Green Master Mix (Promega, Madison, WI, USA) in a T100 thermal cycler (Bio-Rad, Hercules, CA, USA). All primers were obtained from Eurofins Genomics (Ebersberg, Germany).

PCR products were separated on 1% agarose gels and visualized using a gel documentation system (G:BOX Chemi, Syngene, Cambridge, UK). Standard ESBL-producing *E. coli* and *Klebsiella pneumoniae* strains from previous studies were included as positive controls in each PCR run [20, 30].

## RESULTS

### Isolation of *E. coli*

**Figure 1:**
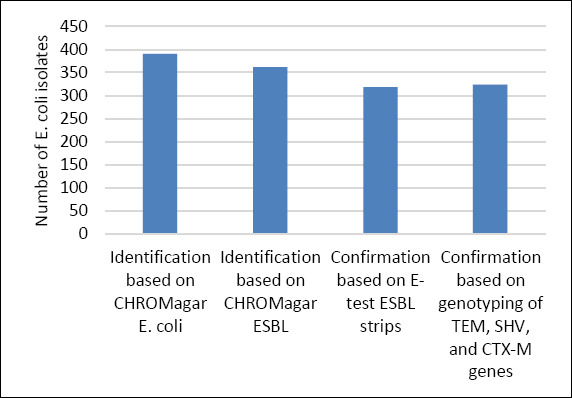
Number of *Escherichia coli* isolates identified using CHROMagar™ *E. coli*, presumptive screening on CHROMagar™ extended-spectrum β-lactamase (ESBL), phenotypic confirmation of ESBL production using E-test ESBL strips, and genotypic confirmation by polymerase chain reaction.

The isolation outcomes for *E. coli* and ESBL-producing *E. coli* based on CHROMagar™ *E. coli* and CHROMagar™ ESBL are presented in Figure 1. Analysis of the 78 examined beef samples yielded 390 *E. coli* isolates showing typical blue colonies on CHROMagar™ *E. coli* (Figure 1). Of these, 361/390 (92.5%) isolates produced dark pink to red colonies after subculture on CHROMagar™ ESBL, indicating presumptive ESBL production (Figure 1).

Number of *Escherichia coli* isolates identified using CHROMagar™ *E. coli*, presumptive screening on CHROMagar™ extended-spectrum β-lactamase (ESBL), phenotypic confirmation of ESBL production using E-test ESBL strips, and genotypic confirmation by polymerase chain reaction.

### Antimicrobial susceptibility profiles of isolates

**Figure 2:**
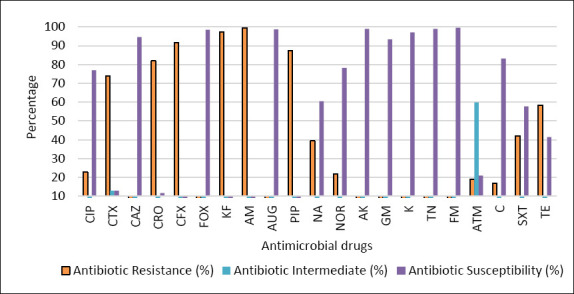
Percentage of resistance, intermediate resistance, and susceptibility of *Escherichia coli* isolates (n = 361) tested against 21 antimicrobial drugs. CIP = Ciprofloxacin, CTX = Cefotaxime, CAZ = Ceftazidime, CRO = Ceftriaxone, CFX = Cephalexin, FOX = Cefoxitin, KF = Cephalothin, AM = Ampicillin, AUG = Augmentin, PIP = Piperacillin, NA = Nalidixic acid, NOR = Noroxin, AK = Amikacin, GM = Gentamicin, K = Kanamycin, TN = Tobramycin, FM = Nitrofurantoin, ATM = Aztreonam, C = Chloramphenicol, SXT = Trimethoprim/sulfamethoxazole, TE = Tetracycline.

The *E. coli* isolates recovered from imported frozen beef and tested against 21 antimicrobial agents exhibited variable susceptibility profiles (Table 1, Figure 2). The highest resistance rates among the 361 isolates were observed for AM (359/361, 99.4%), KF (351/361, 97.2%), CFX (330/361, 91.4%), PIP (315/361, 87.2%), CRO (296/361, 81.9%), and CTX (267/361, 73.9%).

**Table 1:** Overall antibiotic susceptibility of *Escherichia coli* isolates (n = 361).

Antibiotic class	Antibiotic	R (%)	I (%)	S (%)
Cephalosporins	Ciprofloxacin	83 (22.9)	0	278 (77.0)
	Cefotaxime	267 (73.9)	47 (13.0)	47 (13.0)
	Ceftazidime	11 (3.0)	9 (2.4)	341 (94.4)
	Ceftriaxone	296 (81.9)	23 (6.3)	42 (11.6)
	Cephalexin	330 (91.4)	10 (2.7)	21 (5.8)
	Cefoxitin	6 (1.6)	0	355 (98.3)
	Cephalothin	351 (97.2)	7 (1.9)	3 (0.8)
Penicillins	Ampicillin	359 (99.4)	0	2 (0.8)
	Amoxicillin/clavulanic acid	3 (0.8)	2 (0.5)	356 (98.6)
	Piperacillin	315 (87.2)	14 (3.8)	32 (8.8)
Quinolones	Nalidixic acid	142 (39.3)	1 (0.2)	218 (60.3)
	Norfloxacin	79 (21.8)	0	282 (78.1)
Aminoglycosides	Amikacin	3 (0.8)	1 (0.2)	357 (98.8)
	Gentamicin	24 (6.6)	0	337 (93.3)
	Kanamycin	9 (2.4)	2 (0.5)	350 (96.9)
	Tobramycin	2 (0.5)	2 (0.5)	357 (98.8)
Nitrofuran	Nitrofurantoin	2 (0.5)	0	359 (99.4)
Monobactams	Aztreonam	69 (19.1)	216 (59.8)	76 (21.0)
Amphenicol	Chloramphenicol	61 (16.8)	0	300 (83.1)
Sulfonamides	Trimethoprim/sulfamethoxazole	152 (42.1)	0	209 (57.8)
Tetracycline	Tetracycline	211 (58.4)	0	150 (41.5)

R = Resistant, I = Intermediate, S = Susceptible.

Percentage of resistance, intermediate resistance, and susceptibility of *Escherichia coli* isolates (n = 361) tested against 21 antimicrobial drugs. CIP = Ciprofloxacin, CTX = Cefotaxime, CAZ = Ceftazidime, CRO = Ceftriaxone, CFX = Cephalexin, FOX = Cefoxitin, KF = Cephalothin, AM = Ampicillin, AUG = Augmentin, PIP = Piperacillin, NA = Nalidixic acid, NOR = Noroxin, AK = Amikacin, GM = Gentamicin, K = Kanamycin, TN = Tobramycin, FM = Nitrofurantoin, ATM = Aztreonam, C = Chloramphenicol, SXT = Trimethoprim/sulfamethoxazole, TE = Tetracycline.

In contrast, the lowest resistance rates were recorded for TN and FM (0.5% each), followed by AK and AUG (0.8% each) (Table 1). Notably, CAZ and FOX demonstrated comparatively low resistance levels of 3.0% and 1.6%, respectively. The highest proportion of intermediate resistance was unexpectedly detected for ATM (59.8%) (Table 1, Figure 2).

### MDR patterns

MDR, defined as non-susceptibility to at least one agent in three or more antimicrobial classes, was detected in 97.2% (351/361) of *E. coli* isolates. All resistance profiles involved non-susceptibility to more than one antimicrobial agent, except for a single isolate that remained susceptible to all tested drugs. The most extensive MDR profile included resistance to up to 16 antimicrobials (Supplementary Table).

The most frequently observed MDR pattern involved resistance to 6 antimicrobials, accounting for 18.2% (66/361) of isolates, followed by resistance to 12 antimicrobials, accounting for 8.8% (32/361) of isolates. Detailed MDR patterns are summarized in (Supplementary Table).

### Prevalence of ESBL among *E. coli* isolates

**Figure 3:**
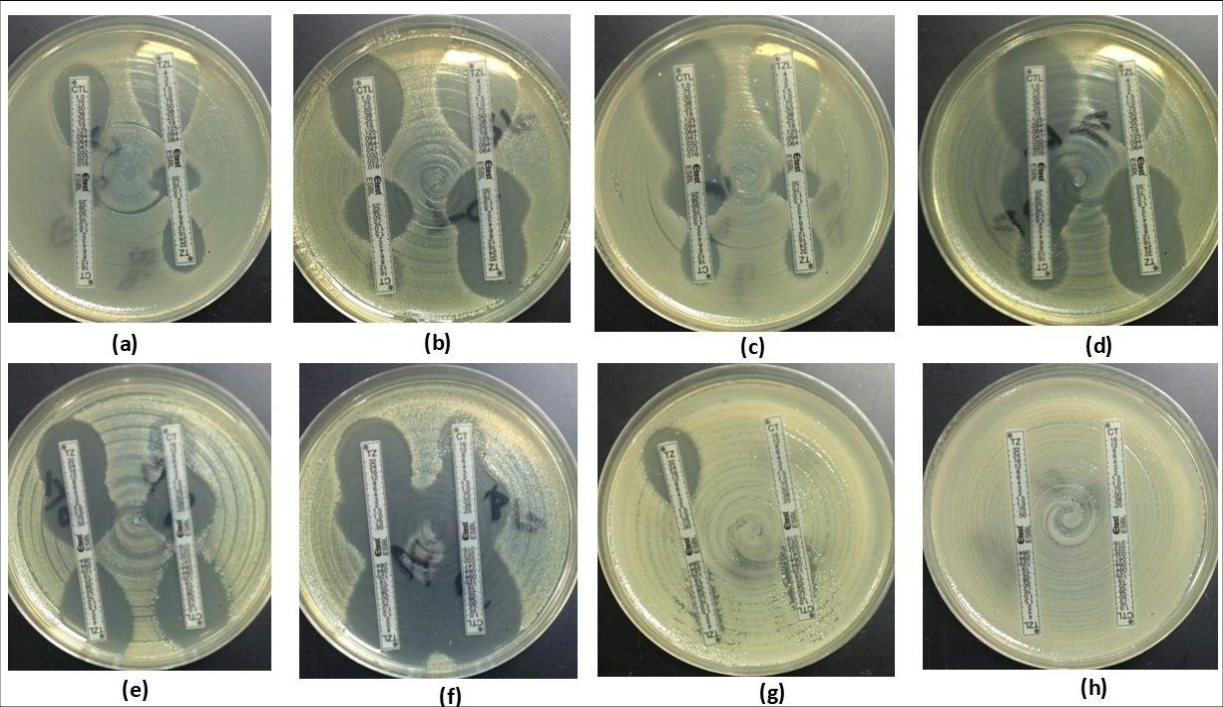
Representative phenotypic patterns of growth inhibition in *Escherichia coli* isolates detected using the extended-spectrum β-lactamase (ESBL) E-test. (a) Distinct ESBL-positive result with a minimum inhibitory concentration (MIC) ratio of cefotaxime/cefotaxime + clavulanic acid (CT/CTL) > 32/0.047 = 32, accompanied by a rounded “phantom” inhibition zone beneath the ceftazidime (TZ) end, indicating ESBL production. (b) Rounded “phantom” inhibition zone beneath the CT end suggestive of ESBL activity, along with distortion of the TZ inhibition ellipse, also consistent with ESBL. (c) Distortion of the CT inhibition ellipse indicative of ESBL, together with a rounded “phantom” inhibition zone beneath the TZ end. (d) Deformation of both CT and TZ inhibition ellipses, characteristic of ESBL. (e) TZ inhibition ellipse deformation combined with a rounded “phantom” inhibition zone beneath the CT end, indicative of ESBL. (f) Deformation observed in both TZ and CT inhibition ellipses, confirming ESBL activity. (g and h) In cases where MIC values exceeded the measurable range of the test device, the results were classified as non-determinable.

**Figure 4:**
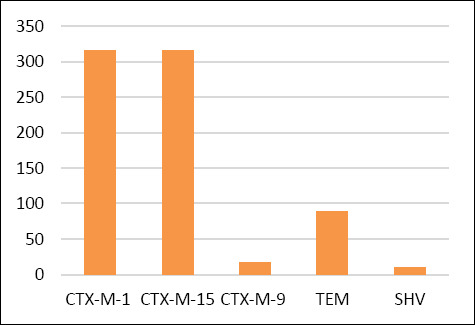
Distribution and occurrence of β-lactamase-encoding genes among *Escherichia coli* isolates (n = 324).

Phenotypic confirmation using E-test ESBL strips demonstrated that 319/361 (88.3%) isolates recovered from CHROMagar™ ESBL exhibited an ESBL-positive phenotype (Figures 1 and 3). Molecular analysis detected β-lactamase-encoding genes in 324/361 (89.7%) isolates. The detected genes included *bla*_TEM_ (n = 10, 2.7%), *bla*_SHV_ (n = 89, 24.6%), *bla*_CTX-M-1_ group (n = 316, 87.5%), *bla*_CTX-M-9_ group (n = 17, 4.7%), and *bla*_CTX-M-15_ (n = 316, 87.5%), as illustrated in Figure 4.

Representative phenotypic patterns of growth inhibition in *Escherichia coli* isolates detected using the extended-spectrum β-lactamase (ESBL) E-test. (a) Distinct ESBL-positive result with a minimum inhibitory concentration (MIC) ratio of cefotaxime/cefotaxime + clavulanic acid (CT/CTL) > 32/0.047 = 32, accompanied by a rounded “phantom” inhibition zone beneath the ceftazidime (TZ) end, indicating ESBL production. (b) Rounded “phantom” inhibition zone beneath the CT end suggestive of ESBL activity, along with distortion of the TZ inhibition ellipse, also consistent with ESBL. (c) Distortion of the CT inhibition ellipse indicative of ESBL, together with a rounded “phantom” inhibition zone beneath the TZ end. (d) Deformation of both CT and TZ inhibition ellipses, characteristic of ESBL. (e) TZ inhibition ellipse deformation combined with a rounded “phantom” inhibition zone beneath the CT end, indicative of ESBL. (f) Deformation observed in both TZ and CT inhibition ellipses, confirming ESBL activity. (g and h) In cases where MIC values exceeded the measurable range of the test device, the results were classified as non-determinable.

Distribution and occurrence of β-lactamase-encoding genes among *Escherichia coli* isolates (n = 324).

## DISCUSSION

### Livestock production and AMR

Livestock species play a vital role in the global food system and contribute substantially to economic, social, and cultural values. Meat remains a nutrient-dense food source worldwide, providing high-quality protein and essential micronutrients [31]. In addition to nutritional importance, livestock production represents a major economic sector in many regions [32]. However, intensive livestock farming faces significant challenges, foremost among them AMR, which poses a serious threat to both human and animal health. The excessive and inappropriate use of antimicrobials in livestock production has accelerated the emergence of resistant bacteria. These bacteria can be transmitted to humans through contaminated meat products, direct animal contact, or environmental pathways [33]. In many production systems, particularly in developing countries, antimicrobials are routinely administered to healthy animals at subtherapeutic doses for disease prevention and growth promotion, creating strong selective pressure for resistance development [34].

### Role of *E. coli* as a foodborne reservoir

Among resistant bacteria, *E. coli* is one of the most prevalent species detected in animal-derived foods. *E. coli* commonly persists in livestock environments, originating from sources such as soil, contaminated feed, water runoff, or heavy metals [35]. Transmission to humans frequently occurs through consumption of cattle and poultry products. Once established in the human gut, resistance determinants are disseminated mainly through horizontal gene transfer. Unregulated antimicrobial use has contributed to the emergence of MDR *E. coli*, with resistance reported against multiple antimicrobial classes, including aminoglycosides, tetracyclines, β-lactams, fluoroquinolones, and third-generation cephalosporins [36–38]. Recent investigations of food-producing animals, including poultry, cattle, and farmed fish, demonstrated moderate to high prevalence of ESBL-producing *E. coli*, with the highest resistance rates consistently observed for TE and AM across geographic regions [38]. These studies also identified pandemic, high-risk zoonotic *E. coli* clones capable of contaminating humans and environmental niches.

### ESBL-producing Enterobacteriaceae and CTX-M dominance

Members of the Enterobacteriaceae family include strains resistant to third-generation cephalosporins, particularly CTX-M group 1 variants, most notably *bla*_CTX-M-1_ and *bla*_CTX-M-15_, which are strongly associated with ESBL production in animals and humans and represent a growing global AMR challenge [38]. The high occurrence of ESBL-producing *E. coli* in imported frozen beef observed in this study highlights the food supply as an important source of MDR bacteria. Such contamination may facilitate transmission through human consumption and underscores the relevance of the One Health framework, linking AMR in livestock to food safety and healthcare systems. Increased prevalence of ESBL-producing Enterobacterales has been widely reported in food and clinical samples worldwide [39–42].

### Molecular characteristics and global comparisons

In the present study, imported frozen beef showed a high prevalence of ESBL-producing *E. coli*, with 88.3% of isolates demonstrating phenotypic ESBL production and 89.7% harboring at least one β-lactamase gene. The most frequently detected genes were *bla*_CTX-M-1_ and *bla*_CTX-M-15_ (each 87.5%), while *bla*_TEM_, *bla*_SHV_, and *bla*_CTX-M-9_ groups were detected at lower frequencies [43–45]. These findings align with reports from Germany, where 93.4% of isolates from dairy and beef cattle carried CTX-M genes, predominantly *bla*_CTX-M-1_ [46]. Similarly, a comprehensive review from Taiwan identified *E. coli* as the most frequently isolated ESBL-producing species in animals, with *bla*_TEM_, *bla*_SHV_, and *bla*_CTX-M_ as the dominant genes [47].

### Public health relevance and One Health perspective

The predominance of CTX-M-1 and CTX-M-15 enzymes among isolates emphasizes the role of broad-spectrum antimicrobial misuse in driving AMR selection and spread [48]. The global expansion of CTX-M-producing *E. coli* has heightened concern regarding zoonotic transmission between animals and humans [39]. The World Health Organization has classified cephalosporin-resistant Enterobacteriaceae as critically important priority pathogens [49], and its Global Action Plan promotes a One Health approach to mitigate AMR [50]. Monitoring resistance markers is essential for identifying emerging trends and assessing health risks. In this study, isolates showed extensive resistance to β-lactams, particularly AM (99.4%), KF (97.2%), and CRO (81.9%), a resistance profile characteristic of ESBL-producing *E. coli* with frequent co-resistance to multiple antimicrobial classes [51]. The MDR rate observed here (97.2%) exceeds that reported in several international retail meat studies [17, 52, 53], underscoring the urgency for stricter antimicrobial regulation in livestock production.

### Antimicrobial use in livestock and global trade implications

Antimicrobials are widely used in livestock farming for disease prevention, treatment, and growth promotion [54]. Their overuse results in environmental residues and sustained selective pressure that favors resistant strains through successive mutation cycles [55]. Approximately half of global antimicrobial production is consumed in livestock, with the United States, China, Australia, and Brazil accounting for 58% of use in 2020 [56]. Antimicrobial consumption is highest in Asia, followed by Oceania and parts of Europe, with TE being the most extensively used agent globally [57]. Such intensive use contributes to the emergence of ESBL-producing bacteria in animal-derived foods [57, 58].

### International trade and dissemination of resistance

The Food and Agriculture Organization has highlighted AMR as a global priority in its action plans [59], noting that countries lacking robust regulatory frameworks are at highest risk. Globalization of the food trade has accelerated cross-border dissemination of AMR, as foods carrying resistance genes on mobile elements are imported into countries with strict domestic regulations [60]. Livestock production in regions with limited oversight, combined with improper waste management, further amplifies environmental dissemination [61]. Similar concerns apply to aquaculture systems operating under weak regulatory conditions [62, 63]. Assessing AMR risks in livestock remains challenging due to limited data on antimicrobial use and the complexity of production systems [62, 63].

### Implications of resistance patterns in imported beef

In this study, resistance among *E. coli* isolates was observed against cephalosporins, penicillins, quinolones, and sulfonamides, with notably high intermediate resistance to ATM (59.8%). Although ATM is primarily reserved for clinical use, reduced susceptibility in foodborne isolates may indicate horizontal gene transfer or co-selection driven by other antimicrobials [64, 65]. Such mechanisms may facilitate the introduction of resistant pathogens into importing countries, including Saudi Arabia. Despite growing evidence of AMR in imported frozen beef and fishery products, data from Saudi Arabia remain limited [20, 36, 64–66]. Imported frozen beef and other animal-derived foods may therefore act as reservoirs for clinically relevant resistance determinants, including ESBLs, plasmid-mediated quinolone resistance, colistin resistance, and carbapenem resistance, posing ongoing risks to the food chain and public health [67].

## CONCLUSION

This study demonstrated an exceptionally high burden of ESBL-producing *E. coli* in retail imported frozen beef, with 88.3% of isolates confirmed phenotypically as ESBL producers and 89.7% harboring at least one β-lactamase gene. The ESBL profile was dominated by *bla*_CTX-M-1_ and *bla*_CTX-M-15_, each detected in 87.5% of isolates, while *bla*_TEM_, *bla*_SHV_, and *bla*_CTX-M-9_ groups were identified at lower frequencies. AST revealed extensive resistance to β-lactams, particularly AM, KF, and CRO, and an alarmingly high MDR rate of 97.2%, with some isolates resistant to up to 16 antimicrobial agents.

The detection of widespread MDR ESBL-producing *E. coli* in imported frozen beef highlights a substantial food safety concern and indicates that international meat supply chains may act as vehicles for the transboundary dissemination of high-risk AMR determinants. The predominance of CTX-M-1 and CTX-M-15 underscores the potential for zoonotic transfer of clinically relevant resistance genes through the food chain. These findings emphasize the need for strengthened import screening, harmonized AMR surveillance, and stricter regulation of antimicrobial use in food-producing animals within a One Health framework.

Key strengths of this study include the integrated use of culture-based isolation, phenotypic ESBL confirmation, comprehensive AST, and molecular genotyping, providing robust and complementary evidence of ESBL and MDR occurrence. The large number of isolates recovered per sample enhanced detection sensitivity, and the focus on imported frozen beef addresses a critical but underexplored AMR pathway in the region.

This study was limited to retail imported frozen beef from a single province, which may not fully reflect national or seasonal variability. Whole-genome sequencing and plasmid characterization were not performed, limiting insights into clonal relatedness, mobile genetic elements, and transmission dynamics. Additionally, quantitative exposure assessment and direct linkage to human infections were beyond the scope of this work.

Future studies should expand surveillance to multiple regions and food matrices, integrate whole-genome sequencing to elucidate transmission pathways, and assess the contribution of specific exporting countries and production systems. Risk assessment studies linking foodborne ESBL-producing *E. coli* to human colonization and infection are also warranted to inform targeted interventions.

Overall, this study provides compelling evidence that imported frozen beef constitutes a significant reservoir of MDR ESBL-producing *E. coli*, predominantly driven by CTX-M-1 and CTX-M-15 enzymes. These findings reinforce the urgency for coordinated One Health strategies, enhanced regulatory oversight, and sustained AMR monitoring to mitigate the public health risks associated with global food trade.

## DATA AVAILABILITY

The supplementary data can be made available from the corresponding author upon request.

## AUTHORS’ CONTRIBUTIONS

LZY: Methodology, data analysis, and drafted and revised the manuscript. NE: Conceptualization, methodology, sample collection, data analysis, and drafted and revised the manuscript. All authors have read and approved the final version of the manuscript.

## COMPETING INTERESTS

The authors declare that they have no competing interests.

## PUBLISHER’S NOTE

Veterinary World remains neutral with regard to jurisdictional claims in the published institutional affiliations.
